# Feasibility and Oncological Safety of Robotic Retroperitoneal Lymph Node Dissection in Patients with Testicular Cancer—Single-Center Experience

**DOI:** 10.3390/cancers17091439

**Published:** 2025-04-25

**Authors:** Markus Angerer, Christian Wülfing, Osama Andura, Mattis Franke, Daniel Robert Stelzl, Klaus-Peter Dieckmann

**Affiliations:** 1 Department of Urology, Asklepios Klinik Altona, Hamburg, 22763 Hamburg, Germany; 2 Department of Urology, University Hospital Hamburg-Eppendorf, 20251 Hamburg, Germany

**Keywords:** testicular cancer, retroperitoneal lymph node dissection, robotic surgery, complication management

## Abstract

**Background/Purpose:** Retroperitoneal lymph node dissection (RPLND) plays a crucial role in the surgical management of testicular cancer. However, RPLND is associated with a high risk of morbidity. Currently, open RPLND (O-RPLND) is considered the gold standard for surgical treatment. The use of minimally invasive techniques has increased significantly over the last few years. This study aimed to compare the safety and oncological outcomes of open (O-RPLND) and robotic (R-RPLND) retroperitoneal lymph node dissection for testicular cancer. **Materials and Methods:** We retrospectively analyzed all the patients who underwent RPLND at our testicular cancer center. Standard O-RPLND was performed with the usual equipment, and R-RPLND was performed with the Da Vinci X/Xi surgical system. The pre- and perioperative parameters and the postoperative complications (Clavien–Dindo classification), anejaculation, and the relapse rate were recorded. The association between the clinicopathological variables and the complications and relapse was assessed using regression analyses. **Results:** Sixty-five patients underwent RPLND during 2017–2024 due to testicular cancer (TC), with thirty-one (47.7%) receiving R-RPLND, including seventeen patients post-chemotherapy (55%). Meanwhile, 34 (52.3%) underwent O-RPLND, comprising 31 patients post-chemotherapy (91%). R-RPLND demonstrated excellent results compared to O-RPLND in terms of the operative time (OT) (*p* < 0.00001). The R-RPLND group had two (6.5%) high-grade (Clavien–Dindo III–V) complications, while four (11.8%) high-grade complications were noted in the O-RPLND group. R-RPLND was linked to a shorter OT (*p* < 0.00001). The hospital stay for R-RPLND was, on average, 2.7 days shorter. In logistic regression analysis, R-RPLND was non-inferior to O-RPLND for overall complications (*p* = 0.6) and low-grade Clavien–Dindo (I-II) (*p* = 0.2) and high-grade Clavien–Dindo (≥III) complications (*p* = 0.7). The median follow-up was 13 months for R-RPLND and 38 months for O-RPLND. Two relapses were observed in the R-RPLND group (6.5%), and two in the O-RPLND group (5.9%). One patient who underwent R-RPLND developed field-edge recurrence. No significant differences in the relapse and anejaculation rates were found between R-RPLND and O-RPLND (*p* = 0.9 and *p* = 0.8, respectively). **Conclusions:** In conclusion, R-RPLND is a feasible procedure with a low complication rate and an acceptable oncological outcome. It has proven to be significantly shorter to O-RPLND in relation to the lengths of HS and OT. However, R-RPLND is a demanding procedure with a considerable learning curve.

## Simple Summary

This study compares open and robotic retroperitoneal lymph node dissection (RPLND) for testicular cancer. The traditional method is open surgery, which carries a high risk of complications. We evaluated these two techniques to assess safety and effectiveness. We found that robotic RPLND results in a shorter operative time and hospital stay and has fewer severe complications, without increasing the risk of recurrence. The aim of this research is to demonstrate that robotic RPLND can be a safer and less-invasive alternative to traditional open surgery. These findings may encourage more surgeons and hospitals to adopt robotic surgery as a standard option, potentially improving recovery and comfort for future patients.

## 1. Introduction

Approximately 50% of nonseminomatous germ cell tumors and 20% of seminomas have metastases at the time of initial presentation [1,2]. The primary mode of metastatic spread in testicular GCTs was established around the turn of 19th and 20th centuries [3]. Retroperitoneal lymph node dissection (RPLND) was developed and promoted by U.S. military surgeons during World War II and immediately thereafter [4,5]. The procedure was shown to be an ideal tool for the clinical staging of patients with testicular germ cell tumors (TGCTs), and the surgical resection of lymph node metastases proved to be of great therapeutic value as well [6,7]. Therefore, RPLND became a standard procedure for treating patients with early-stage nonseminomatous GCTs in the latter half of the last century. However, standard open RPLND (O-RPLND) gradually lost its significance due to increasing concerns about the complications and long-term sequelae associated with the procedure [8,9]. Clearly, standard RPLND represents a major surgery with the risk of complications, such as vascular injury, blood loss, chylous ascites, abdominal organ damage, postoperative ileus, and ureteric lesions. A specific complication is the loss of prograde ejaculation due to the dissection of postganglionic sympathetic nerves in the peri-aortic space [10,11]. Currently, the guidelines recommend RPLND mainly for the resection of post-chemotherapy (pc) residual masses of nonseminomatous GCTs (NSGCTs). In the primary setting, i.e., before any systemic therapy, RPLND is recommended for cases with marker-negative lymphadenopathy or in select instances with clinical stage (CS) one alongside risk factors for progression [12,13,14]. Recently, primary RPLND has also been advocated for cases of pure seminoma exhibiting low-volume retroperitoneal metastases (CS2a,b) [15].

Importantly, during the last decade, RPLND has regained acceptance by the caregivers of patients with a TGCT because of increasing concern about the long-term toxicity of cisplatin-based chemotherapy, particularly the increased rates of second malignancies and of cardiovascular morbidity [16]. The evidence for the persistence of cisplatin in body fluids, for several decades, supported the concerns regarding the uncritical application of chemotherapy in most of patients with a GCT [17].

However, during the last few decades, minimally invasive surgical techniques have become available. Laparoscopic RPLND was the first development utilized by a few experienced surgeons, but it did not gain general acceptance [18]. However, in recent years, other minimally invasive procedures using the Da Vinci robotic system have become the surgical standards in major urologic surgeries, particularly radical prostatectomy, cystectomy, and nephrectomy. In 2006, the first robotic RPLND (R-RPLND) was performed at the Geisinger Medical Center in Pennsylvania [19]. Since then, this new surgical technique has slowly become more popular because RPLND is only required in selected patients with a TGCT and is therefore not a very common procedure in contrast to other urological procedures such as radical prostatectomy. Another impeding factor is the limited availability of the Da Vinci system during the first few years. Currently, robotic operation systems are available in most centers of urologic surgery and in the treatment centers of TGCTs. Accordingly, several reports on R-RPLND have been published documenting the feasibility and efficacy of the method [20,21,22,23,24]. From these reports, it appears conceivable that R-RPLND may open up a new avenue in the management of TGCTs because of the growing concerns about the long-term toxicity of chemotherapy on the one side and the apparently low morbidity of the new procedure on the other side. Therefore, we evaluated our experience with R-RPLND.

Our aim was to show the technical feasibility of the procedure and to provide evidence for its non-inferiority regarding oncological safety by comparing the outcomes of R-RPLND with those of standard O-RPLND.

## 2. Methods

We retrospectively reviewed all the RPLNDs performed at our center between 2017 and 2024 for stage IIA-IIIC GCTs using our institutional electronic patient record system.

R-RPLND was instituted in our department in 2019. During the first year, clinical decision making regarding R-RPLND versus open standard surgery involved some selection towards submitting less-complicated cases to robotic surgery. Since 2020, the majority of cases underwent R-RPLND, with the exception of post-chemotherapy cases with huge or multiple retroperitoneal masses. Overall, there were around 10–12 RPLNDs per year during the study period.

The selection of RPLND and follow-up after surgery was in full accordance with the National and European guidelines [12,13].

The following data were registered in each case: patient demographics (age and BMI), GCT characteristics with histology of orchiectomy specimen and pre-operative staging analysis with largest transverse diameter of retroperitoneal nodes, CS according to the UICC, IGCCCG risk group classification, and previous chemotherapy regimens. With regard to surgical technique, we recorded the mode of surgery (O-RPLND or R-RPLND), the time-point of surgery (primary, i.e., before systemic therapy, or secondary, i.e., pc RPLND), surgical template (unilateral/bilateral), operative time (OT), estimated blood loss (EBL), hospital stay (HS), and final pathology of RPLND specimen stratified as follows: vital GCT other than teratoma [VC]; teratoma or somatic type malignancy [TE]; necrosis/fibrosis [NF]); the number of lymph nodes resected (LNC), and the number of nodes involved with a GCT (“positive nodes” (PN)). Complications within 90 days postoperatively were registered according to the Clavien–Dindo Classification with Grades I–II complications rated as minor/low-grade and Grades IIIa–V as major/high-grade. Follow-up information, particularly the relapse and survival data, was recorded. The follow-up time was calculated from the R-RPLND date until the last clinical follow-up date. All study activities were conducted in compliance with the Helsinki Declaration of the World Medical Association as amended by the 64th General Assembly in 2013. Ethical approval was provided by the Ethikkommission der Ärztekammer Hamburg (PV7288).

### 2.1. Statistical Analysis

The descriptive data are presented as frequencies and median with inter quartile range (IQR). The results for the O-RPLNDs and the R-RPLNDs for various parameters were then compared to each other using a Mann–Whitney test. Regression analysis was used to analyze the risk factors for EBL, DOD, OT, LNC, and PN. Statistical significance in this study was set at *p* < 0.05. R-Studio (Posit Software, PBC formerly RStudio, PBC, Version 2024.09.1+394, 2024, Boston, MA, USA) was used for analysis.

### 2.2. Surgical Technique

O-RPLND was standardized by performing a large midline incision as detailed earlier [25]. All the O-RPLNDs were performed by a team of two surgeons (OA and KPD), while all the R-RPLNDs were conducted by one single surgeon (CW). Abdominal drains were routinely placed only in open RPLND and removed as soon as no chylous fluid was noted after the intake of full oral nutrition. One of the O-RPLNDs was a salvage procedure, but no desperation surgery was performed. The wide cord structures of the tumor side were resected as they were easily accessible, ideally from the internal inguinal ring.

R-RPLND was performed using the DaVinci X/Xi System using a transperitoneal approach with the patient in Trendelenburg position, and the robot was docked over the patient’s head as described previously. Exact port placement depended on the location of the target area. In general, a four-port oblique line placement was used. The camera port (8 mm) was placed in a midline position 4 cm caudal to the umbilicus, and three additional ports (8 mm) were placed in an oblique line, including an assistant port (12 mm). Surgical resection was always template-based.

The right unilateral template is located between the right renal artery and the bifurcation of the right common iliac artery in the cranio-caudal direction and from the right ureter to the mid-aorta in the horizontal direction. The left unilateral template lies between the left renal artery and the bifurcation of the common iliac artery in the cranio-caudal direction and between the left ureter and the mid aorta in the horizontal direction [26]. No re-docking was needed in any of the cases.

Minor refinements of the surgical technique were implemented during the time-course of the study without changing the principal surgical procedure, which resulted in a significant reduction in operating time in the second half of the study period.

## 3. Results

A total of 65 RPLNDs for GCTs were performed between 2017 and March 2024, including 31 R-RPLNDs (47.7%) and 34 O-RPLNDs (52.3%). Of the R-RPLNDs, 17 (55%) were performed in a primary setting, while 14 (45%) were post-chemotherapy resections. O-RPLND was predominantly performed for post-chemotherapy resections (n = 31; 91%), with only three procedures in the primary setting. Two O-RPLNDs were redo-RPLNDs.

### 3.1. Perioperative Outcomes and Complications of R-RPLND Versus O-RPLND

The patients’ clinical and paraclinical parameters are outlined in Table 1, and the postoperative as well as surgical results are outlined in Table 2.

**Figure 1:**
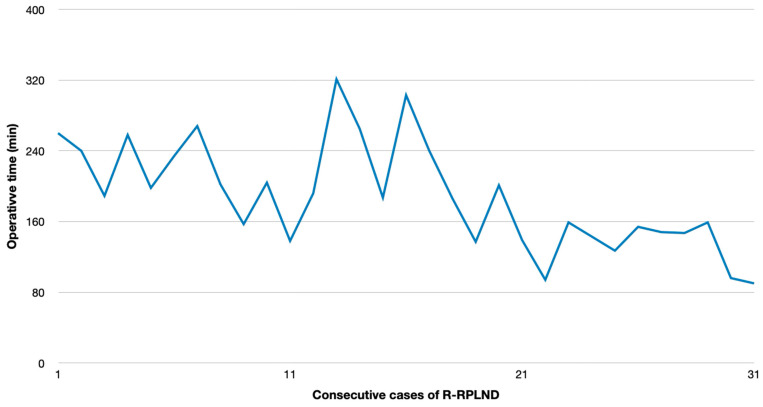
Association of operative time with consecutive case number.

R-RPLND revealed superior results over O-RPLND regarding the OT (*p* < 0.00001). The plotting of the OT versus calendar days since first R-RPLND revealed a considerable learning curve with a significantly shorter operating time in the more recent cases (*p* < 0.001) (Figure 1). The HS for R-RPLND was a total of 2.7 days shorter; however, no statistical significance was observed compared to O-RPLND (*p* = 0.2).

By contrast, O-RPLND involved lymph nodes of larger axial diameters that needed to be resected than those in R-RPLND (2.2 cm vs. 6.4 cm; *p* = 0.0013). LNC was 12.7 vs. 18.22 (*p* = 0.03) higher for O-RPLND. No difference between the two procedures was observed with respect to the number of PNs.

There were two (6.4%) major vascular complications in R-RPLND, both in post-chemotherapy procedures. One patient was converted intraoperatively to an open procedure due to aortic laceration with a major hemorrhage (Clavien–Dindo Grade IIIb). One other required the oversewing of the vena cava intraoperatively (Clavien–Dindo Grade IIIa).

Postoperatively, seven (22.6%) Grade II complications were recorded in the R-RPLND group. One patient required blood transfusion, three had prolonged lymphorrhea (all primary R-RPLND), and two chylorrhoea (one primary and one pcRPLND) and one transient leg paresis cases were documented. No patient needed extra percutaneous drainage.

In the O-RPLND group, four (11.8%) major complications were recorded, one lymphorrea managed with percutaneous drainage (Clavien–Dindo Grade IIIa), one intraoperative vena cava and aorta suture (Clavien–Dindo Grade IIIb), one intraoperative vena cava patch (Clavien–Dindo Grade IIIb), and one intraoperative aortic graft (Clavien–Dindo Grade IIIb). Furthermore, four (11.8%) Grade II complications were encountered, two cases with postoperative blood transfusion, and two with cyhlorrhea requiring intravenous nutrition. In one patient, O-RPLND was discontinued because of unexpected huge inoperable tumor bulk.

There were no Clavien–Dindo Grade 4 or 5 complications among all the RPLND cases. Statistical analysis revealed R-RPLND to be non-inferior to O-RPLND with regard to the overall complications (*p* = 0.6) and also with regard to any particular Clavien–Dindo low-grade (I–II) (*p* = 0.2) or high-grade (*p* = 0.7) complications. In addition, logistic regression analysis did not reveal any particular clinical factor to be associated with the complications. The results of logistic regression are shown in Table 3, Table 4, Table 5, Table 6, Table 7 and Table 8.

### 3.2. Pathological, Oncological, and Functional Outcomes

The median follow-up for R-RPLND was 16 months. Eleven (35%) patients received adjuvant chemotherapy. Two (6.4%) patients recurred following R-RPLND. One patient developed extensive bulky retroperitoneal disease 1 month after R-RPLND. One other patient developed field margin recurrence 13 month after R-RPLND. He underwent open redo-RPLND.

The median follow-up for the O-RPLND group was 38 months. One patient had a fourth retroperitoneal recurrence 3 month after O-RPLND and received radiation therapy. One other had, shortly after O-RPLND, a massive intraperitoneal progression of somatic-type malignancy shortly after O-RPLND.

No significant difference was found between R-RPLND and O-RPLND regarding the relapse rates (Odds Ratio (OR): 0.91; 95% Confidence Interval (CI): (0.12, 6.84)). Logistic regression analysis did not reveal any particular clinical factor to be associated with relapse for R-RPLND and O-RPLND (Table 9 and Table 10).

## 4. Discussion

The crucial result of the present study is that R-RPLND is not inferior to standard O-RPLND with regard to the oncological outcomes, and it is significantly superior to O-RPLND in relation to the lengths of HS and OT. However, R-RPLND is a demanding procedure with a considerable learning curve.

In our series, the mean OT and HS were 189 min and 7.7 days for R-RPLND versus 259 min and 10.4 days for O-RPLND. The enhanced recovery after surgery (ERAS) protocol for O-RPLND was followed, making length-of-stay reduction unlikely. R-RPLND showed a significantly shorter OT (*p* < 0.00001), and the HS was 2.7 days shorter (*p* = 0.2). An increasing caseload linked to a faster OT (*p* < 0.001) suggests potential further decreases within the learning curve. The OT matches Lin and Lloyd et al.’s findings, while Li et al. and Bergdahl et al. reported similar OTs for both the approaches [23,27,28,29]. Notably, robotic surgery had a longer OT compared to that of open surgery for other urological cancers, like radical cystectomy and partial nephrectomy [23,30,31]. Faster patient discharges were noted in several studies and also observed in ours, raising questions about R-RPLND’s economic implications due to its shorter OT and faster discharges [23,27,28,32,33,34]. Nationwide Inpatient Sample analysis reported a median HS of 1.5 days for R-RPLND and 4 days for O-RPLND, highlighting controversies from differing healthcare systems and discharge policies [35].

Furthermore, the postoperative white blood cell count (WBC), considered a biomarker for inflammation, was less elevated, and the levels of red blood cells (RBCs) and hemoglobin (Hb) were higher in the R-RPLND group. This finding was also reported by Lin et al. and is likely due to fewer surgical injuries and less intraoperative fluid loss, which may contribute to faster postoperative recovery [29]. The amount of EBL was significantly lower (*p* < 0.00001) in R-RPLND, but similar blood transfusion rates were observed. Few studies have reported on these rates, and three found significantly fewer transfusions in patients who underwent R-RPLND (0.9% vs. 14.5% for O-RPLND; *p* < 0.001) [36].

The preoperative tumor mass was 2.2 cm vs. 6.4 cm for R-RPLND and O-RPLND (*p* = 0.0013), but despite this considerable difference in tumor bulk, our series saw no significance between R-RPLND vs. O-RPLND for the LNC (*p* = 0.03) or the PN (*p* = 0.733). Three studies compared the lymph node yield between R-RPLND and O-RPLND, but did not detect significant differences. Lin et al. noted R-RPLND retrieved more lymph nodes, correlating with a better prognosis. The total lymph node count depends on the surgical approach, patient variability, specimen labeling (en bloc vs. packet), and pathological processing. The complete removal of the lymph node packet is crucial for superior disease-free survival [29,36].

The complication rate (overall complication and low-grade or high-grade complication) for R-RPLND was not significantly different from that of the O-RPLND group (*p* = 0.6, *p* = 0.2, *p* = 0.7, respectively). The most common complication noted was CA, which requires attention. For all the R-RPLND procedures, no extra percutaneous drainage was necessary. Additionally, logistic regression analysis for R-RPLND and O-RPLND indicated no predictive factors for complications.

The previous studies have indicated that R-RPLND demonstrated significantly lower overall complication rates in comparison to those of O-RPLND. Furthermore, these investigations reported comparable rates of major (Grade ≥ III) complications between the two procedural groups. Nason et al. reported a low complication rate for R-RPLND (3.7% minor complications; 11.1% major complications). We have noted two major complications (6.5%) in post-chemotherapy (pc) R-RPLND. Blok et al. showed a 4.4% incidence of high-grade complications for pcR-RPLND. A meta-analysis by Yuan et al. and Ge et al. demonstrated that patients with R-RPLND exhibited a significantly lower incidence of total complications than the non-robotic RPLND group (*p* = 0.002 and *p* < 0.05). However, for Clavien–Dindo Grade ≥ III complications, no statistically significant distinction was found between the two groups (*p* = 0.08 and *p* > 0.05) [33,34,37,38]. Postoperative ejaculation disorders were observed in 25.1% and 17.7% of R-RPLND and O-RPLND, respectively. However, a nerve-sparing technique was not attempted in cases with huge and multiple masses to not compromise the oncological outcome.

Few studies have reported oncological outcomes. The present study reports two recurrences after O-RPNLD (5.9%), and also after R-RPLND (6.4%). No statistically significant difference (*p* = 0.9) in the relapse rates was observed between the groups in this series. All the consecutive R-RPLND cases were included in analysis, thus the learning curve was not a determining factor for the tumor recurrence rate. Grenabo Bergdahl et al. also reported comparable disease recurrence rates of 8.6% after O-RPLND and 6.8% after R-RPLND, consistent with the present series [27]. Li et al. observed similar relapse rates for R-RPLND (10%) (vs. O-RPLND 19%) [26]. Conversely, Lloyd et al. found no tumor recurrence for O-RPLND and R-RPLND (median follow-up 36 months), suggesting R-RPLND is oncologically safe in the short term [20]. Recently, Calaway et al. reported adverse surgical outcomes associated with R-RPLND. One patient experienced in-field recurrence, and four patients exhibited out-of-field recurrence in atypical locations. The recurrence patterns following R-RPLND deviated from the previous experiences managing recurrences after open RPLND. The treatment burden was substantial, necessitating various chemotherapy regimens, while acknowledge the unknown number of patients successfully treated with R-RPLND [39].

Pearce et al. reported the early outcomes of 47 primary RPLNDs. Despite the predominant early stage, they reported one out-of-field recurrence with a 2-year recurrence-free survival of 97% [36]. Rocco et al. reported 58 primary R-RPLNDs with a 4-year follow-up. The 2-year recurrence-free survival was 91%, with five patients developing out-of-field recurrences [37]. After a median follow-up of over 3 years, none of the patients exhibited evidence of the disease after pc R-RPLND, as reported by Blok et al. [35]. A meta-analysis by Yuan et al. demonstrated that the difference in recurrence rates between R-RPLND and O-RPLND was not statistically significant (*p*= 0.46) [29].

After the first R-RPLND in 2006, several comparisons have proved its consistency in oncological outcomes with those of standard O-RPLND [19,22,29,36,37]. Meanwhile, R-RPLND demonstrated its superiority over O-RPLND in the perioperative outcomes, especially the OT, EBL, and the HS [23,29]. Additionally, R-RPLND is a de-escalating approach, aiming to maintain the traditional excellent oncologic result, while minimizing the treatment burden and toxicity [13]. Chemotherapy is effective, but the side effects of chemotherapeutic agents and the risks of cardiovascular disease and secondary tumors are challenging to overcome [33]. The potential hazards of chemotherapy also make RPLND a more acceptable option [29].

The present study revealed both R-RPLND and O-RPLND to be safe and efficacious for the purpose of retroperitoneal lymph node dissections in patients with testis cancer. The oncological outcomes, the frequency of relapses, the number of complications, and the lymph node yields were quite similar with both the surgical methods. But of note, by comparison, R-RPLND offered a significantly reduced OT, a shorter HS, and faster recovery.

These results underscore the remarkable advantages of R-RPLND in minimizing the risks associated with the procedure [33]. The current literature indicates that R-RPLND is both safe and feasible in the primary and post-chemotherapy settings [28,37,38]. Maintaining oncological efficacy is an important prerequisite for the adoption of a minimally invasive approach because the patients are relatively young, and long-term survival is expected in most cases [38].

Our study is not without limitations. The major limitation is its retrospective nature, introducing analytical and selection biases, as the patients chosen for R-RPLND may have had more favorable disease characteristics, smaller tumors, and a lower disease risk. Despite the small sample size, the fact that most patients had viable tumors lends significance to our findings on the oncological effectiveness of R-RPLND. Additionally, we included all the patients treated for RPLDN at our testicular cancer center in our analysis. Furthermore, our series included patients who underwent pcR-RPLND, but primary vs. pc-RPLND subanalysis was performed.

The comparison of the long-term sequelae of the two procedures is hampered by the considerably shorter period of follow-up in the R-RPLND cases (16 months versus 38 months in O-RPLND).

The finding that the frequencies of complications and of relapse are not associated with the covariates must be considered with caution because the small sample size might have influenced this result.

R-RPLND shows promising potential and is a safe, feasible, and efficacious treatment option for metastatic TC. The optimal selection criteria for O-RPLND and R-RPLND are yet to be defined [36]. To date, R-RPLDN should be restricted to high-volume centers with expertise in open and R-RPLND [28]. Further research, especially prospective randomized trials, is needed to confirm these findings and assess the true efficacy and oncological safety of R-RPLND, as well as further investigation into the optimal patient selection for R-RPLND. Specifically, the long-term outcomes remain limited. Therefore, R-RPLND may be an appropriate treatment option.

## 5. Conclusions

R-RPLND demonstrates promising potential as a safe and effective treatment for TC. R-RPLND exhibits comparable outcomes to those of open procedures in selected cases with low-volume residual disease and when performed by highly experienced surgeons.

This study elucidates the key advantages of R-RPLND, including a reduced OT and expedited recovery. R-RPLND has demonstrated short-term oncological efficacy and may potentially reduce the necessity for adjuvant chemotherapy. These benefits render R-RPLND a valuable option with encouraging initial outcomes. This approach warrants consideration in specialist TC centers with expertise in O-RPLND and minimally invasive surgery to ensure appropriate case selection. Future research should focus on larger multicenter prospective studies with extended follow-up durations and randomized controlled trials to establish a more comprehensive understanding of the role of R-RPLND.

In conclusion, R-RPLND is both safe and feasible in primary and post-chemotherapy settings, demonstrating acceptable perioperative and short-term oncological outcomes.

## Figures and Tables

**Table 1:** Patients’ clinical and paraclinical parameters.

	R-RPLND (n = 31)	O-RPLND (n = 34)	*p*-Value ^1^
**Age (mean, years)**	33.90	37.90	<0.00001
**BMI**	26.62	26.27	0.1
**Preoperative tumor mass (mean, cm)**	2.19	6.38	0.0013
**Prior abdominal operations, n (%)**			0.8
y	4 (12.9%)	4 (11.8%)	
n	27 (87.1%)	30 (88.2%)	
**Post-chemotherapy, n (%)**			0.00001
y	11 (35.5%)	31 (91.2%)	
n	20 (64.5%)	3 (8.8%)	
**Surgical indication, n (%)**			0.00024
Primary	18 (58.1%)	4 (11.8%)	
Secondary	13 (41.9%)	30 (88.2%)	
**Prognostic group (IGCCCG), n (%)**			0.0025
good prognosis	30 (96.8%)	21 (61.8%)	
intermediate prognosis	1 (3.2%)	7 (20.6%)	
poor prognosis	0 (0.0%)	6 (17.8%)	
**Histology at orchiectomy, n (%)**			0.08
nonseminoma	25 (80.7%)	33 (97.1%)	
Seminoma	6 (19.4%)	1 (2.9%)	
Biopsy retroperitoneal	0 (0.0%)	1 (2.9%)	
**Preoperative paraclinical parameters (mean)**			
LDH (U/L)	199.67	197.46	0.08
ß-HCG (U/L)	1.43	1.41	0.9
AFP (ug/L)	3.84	3.72	0.9
Hb (g/dL)	14.45	13.69	0.5
TSH (mU/L)	1.47	1.74	0.8
RBC (n/pl)	4.77	4.66	0.9
WBC (n/nl)	6.23	6.70	0.7
Kreatinin (mg/dL)	0.97	0.90	0.9

^1^ *t*-Test, Chi square test or Fishers exact test; y = yes; n = no; Hb = hemoglobin; WBC = white blood cell; RBC = red blood cell.

**Table 2:** Postoperative results.

	R-RPLND (n = 31)	O-RPLND (n = 34)	*p*-Value ^1^
**OT (mean, min)**	189.43	259.32	<0.00001
**EBL (mean, ml)**	226.43	430.00	<0.00001
**HS (mean, days)**	7.79	10.42	0.2
**postoperative paraclinical parameters (mean)**			
Hb (g/dL)	12.43	11.46	0.6
Hb (g/dL)	1.32	1.79	0.8
RBC (n/pl)	4.14	3.77	0.8
RBC (n/pl)	8.56	11.48	0.2
Kreatinin (mg/dL)	0.92	1.13	0.9
**Type of Complication, n (%)**			0.3
Intraoperative	2 (6.5%)	4 (11.8%)	
Postoperative	6 (19.4%)	2 (5.9%)	
**Overall complications, n (%)**			0.6
y	8 (25.8%)	6 (17.6%)	
n	23 (74.2%)	28 (82.3%)	
**Clavien–Dindo I-II, n (%)**			0.2
y	6 (19.4%)	2 (5.9%)	
n	25 (80.7%)	32 (94.1%)	
**Clavien–Dindo ≥ III, n (%)**			0.7
y	2 (6.5%)	4 (11.8%)	
n	29 (93.5%)	30 (88.2%)	
**Relapse, n (%)**			0.9
y	2 (6.5%)	2 (5.9%)	
n	29 (93.5%)	32 (94.1%)	
**Final Pathology, n (%)**			0.8
Viable tumor	26 (83.9%)	27 (79.4%)	
Necrosis/no malignancy	5 (16.1%)	7 (20.6%)	
**Lymph node count (mean, n)**	12.70	18.22	0.03
**Positive lymph nodes (mean, n)**	2.52	1.67	0.7
**Lymph node positivity rate (mean, %)**	22.6	15.3	0.05
**Ejaculation disorder, n (%)**			0.4
y	8 (25.81%)	6 (17.65%)	
n	23 (74.19%)	28 (82.35%)	
**Anejaculation, n (%)**			0.8
y	4 (12.90%)	5 (14.71%)	
n	27 (87.10%)	29 (85.29%)	
**Retrograde ejaculation, n (%)**			
y	4 (12.90%)	1 (2.94%)	0.15
n	27 (87.10%)	33 (97.06%)	
**Follow-up (median, month)**			
	16	38	<0.00001

^1^ *t*-Test, Chi square test or Fishers exact test; y = yes; n = no; Hb = hemoglobin; WBC = white blood cell; RBC = red blood cell.

**Table 3:** Association of overall complications for R-RPLND as per regression analysis.

Univariate Analysis				Multivariate Analysis			
Variable	OR ^1^	95% CI ^2^	*p*-Value	Variable	OR ^1^	95% CI ^2^	*p*-Value
**Case Number**	1.02	0.95–1.10	0.5	**Case Number**	1.01	0.95–1.10	0.5
**BMI**	0.98	0.81–0.98	0.8	**BMI**	0.98	0.81–0.98	0.8
**Age**	1.02	0.88–1.18	0.7	**Age**	1.02	0.88–1.18	0.7
**Prior abdominal operations**	0.95	0.08–10.7	0.9	**Prior abdominal operations**	0.95	0.08–10.7	0.9
**Clinical stage (UICC)**				**Clinical stage (UICC)**			
IIA	3.51	0.40–30.3	0.2	IIA	1.53	0.048–52.1	0.8
IIB	0.19	0.02–1.76	0.4	IIB	0.94	1.01–8.32	0.3
IIC	-	-	1.0	**Prognostic group (IGCCCG)**			
III	-	-	1.0	GP	1.90	0.32–11.20	0.4
**Prognostic group (IGCCCG)**				IP	2.40	0.34–16.90	0.3
GP	1.90	0.32–11.20	0.4	**post-chemotherapy (y)**	0.38	0.006–24.0	0.6
IP	2.40	0.34–16.90	0.3	**Preoperative tumor mass**	0.98	0.08–1.19	0.5
PP	-	-	1.0	**OT**	1.05	0.99–1.01	0.1
**post-chemotherapy (y)**	3.50	0.40–30.3	0.2	**EBL**	1.01	0.99–1.01	0.1
**Preoperative tumor mass**	0.98	0.08–1.19	0.5	**LNC**	0.95	0.81–1.12	0.5
**OT**	1.05	0.99–1.01	0.1	**PN**	0.68	0.29–1.59	0.3
**EBL**	1.00	1.00–1.00	0.1	**Final histology (viable tumor)**	2.22	0.004–1165.5	0.8
**LNC**	0.95	0.81–1.12	0.5				
**PN**	0.70	0.42–1.15	0.1				
**Final histology (viable tumor)**	1.58	0.23–10.9	0.6				

^1^ OR = Odds Ratio, ^2^ CI = Confidence Interval, OT = operative time, EBL = estimated blood loss, LNC = lymph node count, PN = positive node, y = yes.

**Table 4:** Association of overall complications for O-RPLND as per regression analysis.

Univariate Analysis				Multivariate Analysis			
Variable	OR ^1^	95% CI ^2^	*p*-Value	Variable	OR ^1^	95% CI ^2^	*p*-Value
**Case Number**	1.02	0.95–1.10	0.5	**Case Number**	1.01	0.95–1.10	0.5
**BMI**	0.98	0.81–0.98	0.8	**BMI**	0.98	0.81–0.98	0.8
**Age**	1.02	0.88–1.18	0.7	**Age**	1.02	0.88–1.18	0.7
**Prior abdominal operations**	0.95	0.08–10.7	0.9	**Prior abdominal operations**	0.95	0.08–10.7	0.9
**Clinical stage (UICC)**				**Clinical stage (UICC)**			
IIA	2.51	0.40–30.3	0.2	IIA	1.53	0.048–52.1	0.8
IIB	0.19	0.02–1.76	0.4	IIB	0.94	1.01–8.32	0.3
IIC	-	-	1.0	**Prognostic group (IGCCCG)**			
III	-	-	1.0	GP	1.90	0.32–11.20	0.4
**Prognostic group (IGCCCG)**				IP	2.40	0.34–16.90	0.3
GP	1.90	0.32–11.20	0.4	**post-chemotherapy (y)**	0.38	0.006–24.0	0.6
IP	2.40	0.34–16.90	0.3	**Preoperative tumor mass**	0.98	0.08–1.19	0.5
PP	-	-	1.0	**OT**	1.05	0.99–1.01	0.1
**post-chemotherapy (y)**	3.50	0.40–30.3	0.2	**EBL**	1.01	0.99–1.01	0.1
**Preoperative tumor mass**	0.98	0.08–1.19	0.5	**LNC**	0.95	0.81–1.12	0.5
**OT**	1.05	0.99–1.01	0.1	**PN**	0.68	0.29–1.59	0.3
**EBL**	1.00	1.00–1.00	0.1	**Final histology (viable tumor)**	2.22	0.004–1165.5	0.8
**LNC**	0.95	0.81–1.12	0.5				
**PN**	0.70	0.42–1.15	0.1				
**Final histology (viable tumor)**	1.58	0.23–10.9	0.6				

^1^ OR = Odds Ratio, ^2^ CI = Confidence Interval, OT = operative time, EBL = estimated blood loss, LNC = lymph node count, PN = positive node, y = yes.

**Table 5:** Association of low-grade complications (Clavien–Dindo I–II) for R-RPLND as per regression analysis.

Univariate Analysis				Multivariate Analysis			
Variable	OR ^1^	95% CI ^2^	*p*-Value	Variable	OR ^1^	95% CI ^2^	*p*-Value
**Case Number**	1.01	0.82–1.23	0.9	**Case Number**	1.01	0.82–1.23	0.9
**BMI**	1.01	0.82–1.23	0.9	**BMI**	1.01	0.82–1.23	0.9
**Age**	1.02	0.88–1.18	0.7	**Age**	1.02	0.88–1.18	0.7
**Prior abdominal operations**	1.46	0.12–17.2	0.7	**Prior abdominal operations**	1.46	0.12–17.2	0.7
**Clinical stage (UICC)**				**Clinical stage (UICC)**			
IIA	1.51	0.12–17.3	0.7	IIA	1.51	0.12–17.3	0.7
IIB	0.30	0.03–2.96	0.3	IIB	0.30	0.0012–8.1	0.4
**Prognostic group (IGCCCG)**				**Prognostic group (IGCCCG)**			
GP	1.75	0.10–30.59	0.7	GP	1.75	0.10–30.59	0.7
IP	-	-	0.99	IP	-	-	0.99
PP	-	-	1.0	**post-chemotherapy (y)**	0.88	0.13–5.84	0.9
**post-chemotherapy (y)**	0.88	0.13–5.84	0.9	**Preoperative tumor size**	0.59	0.82–1.23	0.3
**Preoperative tumor size**	0.59	0.82–1.23	0.3	**OT**	1.00	0.99–1.00	0.9
**OT**	1.00	0.99–1.00	0.9	**EBL**	1.00	0.99–1.00	0.9
**EBL**	1.00	0.99–1.00	0.9	**LNC**	0.94	0.79–1.13	0.5
**LNC**	0.94	0.79–1.13	0.5	**PN**	0.82	0.51–1.33	0.4
**PN**	0.82	0.51–1.33	0.4	**Final histology (viable tumor)**	0.95	0.087–10.4	0.9
**Final histology (viable tumor)**	0.95	0.087–10.4	0.9				

^1^ OR = Odds Ratio, ^2^ CI = Confidence Interval, OT = operative time, EBL = estimated blood loss, LNC = lymph node count, PN = positive node, y = yes.

**Table 6:** Association of low-grade complications (Clavien–Dindo I–II) for O-RPLND as per regression analysis.

Univariate Analysis				Multivariate Analysis			
Variable	OR ^1^	95% CI ^2^	*p*-Value	Variable	OR ^1^	95% CI ^2^	*p*-Value
**Case Number**	1.01	0.82–1.23	0.9	**Case Number**	1.01	0.82–1.23	0.9
**BMI**	1.01	0.82–1.23	0.9	**BMI**	1.01	0.82–1.23	0.9
**Age**	1.02	0.88–1.18	0.7	**Age**	1.02	0.88–1.18	0.7
**Prior abdominal operations**	1.46	0.12–17.2	0.7	**Prior abdominal operations**	1.46	0.12–17.2	0.7
**Clinical stage (UICC)**				**Clinical stage (UICC)**			
IIA	1.51	0.12–17.3	0.7	IIA	1.51	0.12–17.3	0.7
IIB	0.30	0.03–2.96	0.3	IIB	0.30	0.0012–8.1	0.4
**Prognostic group (IGCCCG)**				**Prognostic group (IGCCCG)**			
GP	1.75	0.10–30.59	0.7	GP	1.75	0.10–30.59	0.7
IP	-	-	0.99	IP	-	-	0.99
PP	-	-	1.0	**post-chemotherapy (y)**	0.88	0.13–5.84	0.9
**post-chemotherapy (y)**	0.88	0.13–5.84	0.9	**Preoperative tumor mass**	0.59	0.82–1.23	0.3
**Preoperative tumor mass**	0.59	0.82–1.23	0.3	**OT**	1.00	0.99–1.00	0.9
**OT**	1.00	0.99–1.00	0.9	**EBL**	1.00	0.99–1.00	0.9
**EBL**	1.00	0.99–1.00	0.9	**LNC**	0.94	0.79–1.13	0.5
**LNC**	0.94	0.79–1.13	0.5	**PN**	0.82	0.51–1.33	0.4
**PN**	0.82	0.51–1.33	0.4	**Final histology (viable tumor)**	0.95	0.087–10.4	0.9
**Final histology (viable tumor)**	0.95	0.087–10.4	0.9				

^1^ OR = Odds Ratio, ^2^ CI = Confidence Interval, OT = operative time, EBL = estimated blood loss, LNC = lymph node count, PN = positive node, y = yes.

**Table 7:** Association of high-grade complications (Clavien–Dindo ≥ III) for R-RPLND as per regression analysis.

Univariate Analysis				Multivariate Analysis			
Variable	OR ^1^	95% CI ^2^	*p*-Value	Variable	OR ^1^	95% CI ^2^	*p*-Value
**Case Number**	1.02	0.95–1.10	0.5	**Case Number**	1.01	0.95–1.10	0.5
**BMI**	0.98	0.81–0.98	0.8	**BMI**	0.98	0.81–0.98	0.8
**Age**	1.02	0.88–1.18	0.7	**Age**	1.02	0.88–1.18	0.7
**Prior abdominal operations**	0.95	0.08–10.7	0.9	**Prior abdominal operations**	0.95	0.08–10.7	0.9
**Clinical stage (UICC)**				**Clinical stage (UICC)**			
IIA	3.51	0.40–30.3	0.2	IIA	1.53	0.048–52.1	0.8
IIB	0.19	0.02–1.76	0.4	IIB	0.94	1.01–8.32	0.3
IIC	-	-	1.0	**Prognostic group (IGCCCG)**			
III	-	-	1.0	GP	1.90	0.32–11.20	0.4
**Prognostic group (IGCCCG)**				IP	2.40	0.34–16.90	0.3
GP	1.90	0.32–11.20	0.4	**post-chemotherapy (y)**	0.38	0.006–24.0	0.6
IP	2.40	0.34–16.90	0.3	**Preoperative tumor mass**	0.98	0.08–1.19	0.5
PP	-	-	1.0	**OT**	1.05	0.99–1.01	0.1
**post-chemotherapy (y)**	3.50	0.40–30.3	0.2	**EBL**	1.01	0.99–1.01	0.1
**Preoperative tumor mass**	0.98	0.08–1.19	0.5	**LNC**	0.95	0.81–1.12	0.5
**OT**	1.05	0.99–1.01	0.1	**PN**	0.68	0.29–1.59	0.3
**EBL**	1.00	1.00–1.00	0.1	**Final histology (viable tumor)**	2.22	0.004–1165.5	0.8
**LNC**	0.95	0.81–1.12	0.5				
**PN**	0.70	0.42–1.15	0.1				
**Final histology (viable tumor)**	1.58	0.23–10.9	0.6				

^1^ OR = Odds Ratio, ^2^ CI = Confidence Interval, OT = operative time, EBL = estimated blood loss, LNC = lymph node count, PN = positive node, y = yes.

**Table 8:** Association of high-grade complications (Clavien–Dindo ≥ III) for O-RPLND as per regression analysis.

Univariate Analysis				Multivariate Analysis			
Variable	OR ^1^	95% CI ^2^	*p*-Value	Variable	OR ^1^	95% CI ^2^	*p*-Value
**Case Number**	1.02	0.95–1.10	0.5	**Case Number**	1.01	0.95–1.10	0.5
**BMI**	0.98	0.81–0.98	0.8	**BMI**	0.98	0.81–0.98	0.8
**Age**	1.02	0.88–1.18	0.7	**Age**	1.02	0.88–1.18	0.7
**Prior abdominal operations**	0.95	0.08–10.7	0.9	**Prior abdominal operations**	0.95	0.08–10.7	0.9
**Clinical stage (UICC)**				**Clinical stage (UICC)**			
IIA	3.51	0.40–30.3	0.2	IIA	1.53	0.048–52.1	0.8
IIB	0.19	0.02–1.76	0.4	IIB	0.94	1.01–8.32	0.3
IIC	-	-	1.0	**Prognostic group (IGCCCG)**			
III	-	-	1.0	GP	1.90	0.32–11.20	0.4
**Prognostic group (IGCCCG)**				IP	2.40	0.34–16.90	0.3
GP	1.90	0.32–11.20	0.4	**post-chemotherapy (y)**	0.38	0.006–24.0	0.6
IP	2.40	0.34–16.90	0.3	**Preoperative tumor mass**	0.98	0.08–1.19	0.5
PP	-	-	1.0	**OT**	1.05	0.99–1.01	0.1
**post-chemotherapy (y)**	3.50	0.40–30.3	0.2	**EBL**	1.01	0.99–1.01	0.1
**Preoperative tumor mass**	0.98	0.08–1.19	0.5	**LNC**	0.95	0.81–1.12	0.5
**OT**	1.05	0.99–1.01	0.1	**PN**	0.68	0.29–1.59	0.3
**EBL**	1.00	1.00–1.00	0.1	**Final histology (viable tumor)**	2.22	0.004–1165.5	0.8
**LNC**	0.95	0.81–1.12	0.5				
**PN**	0.70	0.42–1.15	0.1				
**Final histology (viable tumor)**	1.58	0.23–10.9	0.6				

^1^ OR = Odds Ratio, ^2^ CI = Confidence Interval^,^ OT = operative time, EBL = estimated blood loss, LNC = lymph node count, PN = positive node, y = yes.

**Table 9:** Association of relapse for R-RPLND as per regression analysis.

Univariate Analysis				Multivariate Analysis			
Variable	OR ^1^	95% CI ^2^	*p*-Value	Variable	OR ^1^	95% CI ^2^	*p*-Value
**Case Number**	1.02	0.95–1.10	0.5	**Case Number**	1.01	0.95–1.10	0.5
**BMI**	0.98	0.81–0.98	0.8	**BMI**	0.98	0.81–0.98	0.8
**Age**	1.02	0.88–1.18	0.7	**Age**	1.02	0.88–1.18	0.7
**Prior abdominal operations**	0.95	0.08–10.7	0.9	**Prior abdominal operations**	0.95	0.08–10.7	0.9
**Clinical stage (UICC)**				**Clinical stage (UICC)**			
IIA	3.51	0.40–30.3	0.2	IIA	1.53	0.048–52.1	0.8
IIB	0.19	0.02–1.76	0.4	IIB	0.94	1.01–8.32	0.3
IIC	-	-	1.0	**Prognostic group (IGCCCG)**			
III	-	-	1.0	GP	1.90	0.32–11.20	0.4
**Prognostic group (IGCCCG)**				IP	2.40	0.34–16.90	0.3
GP	1.90	0.32–11.20	0.4	**post-chemotherapy (y)**	0.38	0.006–24.0	0.6
IP	2.40	0.34–16.90	0.3	**Preoperative tumor mass**	0.98	0.08–1.19	0.5
PP	-	-	1.0	**OT**	1.05	0.99–1.01	0.1
**post-chemotherapy (y)**	3.50	0.40–30.3	0.2	**EBL**	1.01	0.99–1.01	0.1
**Preoperative tumor mass**	0.98	0.08–1.19	0.5	**LNC**	0.95	0.81–1.12	0.5
**OT**	1.05	0.99–1.01	0.1	**PN**	0.68	0.29–1.59	0.3
**EBL**	1.00	1.00–1.00	0.1	**Final histology (viable tumor)**	2.22	0.004–1165.5	0.8
**LNC**	0.95	0.81–1.12	0.5				
**PN**	0.70	0.42–1.15	0.1				
**Final histology (viable tumor)**	1.58	0.23–10.9	0.6				

^1^ OR = Odds Ratio, ^2^ CI = Confidence Interval, OT = operative time, EBL = estimated blood loss, LNC = lymph node count, PN= positive node, y = yes.

**Table 10:** Association of relapse for O-RPLND as per regression analysis.

Univariate Analysis				Multivariate Regression Results			
Variable	OR ^1^	95% CI ^2^	*p*-Value	Variable	OR ^1^	95% CI ^2^	*p*-Value
**Case Number**	1.02	0.95–1.10	0.5	**Clinical stage (UICC)**			
**BMI**	0.98	0.81–1.19	0.8	- IIA	1.53	0.048–52.1	0.8
**Age**	1.01	0.95–1.08	0.7	- IIB	0.94	1.01–8.32	0.3
**Prior abdominal operations**	0.95	0.08–10.7	0.9	**post-chemotherapy (y)**	0.38	0.006–24.0	0.6
**Clinical stage (UICC)**				**PN**	0.68	0.29–1.59	0.3
IIA	3.51	0.40–30.3	0.2	**Preoperative tumor mass**	1.01	0.99–1.01	0.1
IIB	0.19	0.02–1.76	0.4	**OT**	1.01	0.99–1.01	0.1
IIC	-	-	1.0	**EBL**	1.00	0.99–1.01	0.9
III	-	-	1.0				
**Prognostic group (IGCCCG)**							
GP	1.00	–	-				
IP	1.50	0.20–11.2	0.7				
PP	1.20	0.15–9.80	0.8				
**post-chemotherapy (y)**	3.50	0.40–30.3	0.2				
**Preoperative tumor mass**	0.98	0.08–1.19	0.5				
**OT**	1.05	0.99–1.01	0.1				
**EBL**	1.00	0.99–1.01	0.9				
**LNC**	0.95	0.81–1.12	0.5				
**PN**	0.70	0.42–1.15	0.1				
**Final histology (viable tumor)**	1.20	0.15–9.80	0.8				

^1^ OR = Odds Ratio, ^2^ CI = Confidence Interval, OT = operative time, EBL = estimated blood loss, LNC = lymph node count, PN = positive node, y = yes.

## Data Availability

The original contributions presented in this study are included in this article. Further inquiries can be directed to the corresponding author(s).
